# The role of occupational therapy in supporting parenting occupations: An Irish occupational therapy perspective

**DOI:** 10.1177/03080226261418491

**Published:** 2026-02-15

**Authors:** Yvonne Codd, Anne Honey, Margaret McGrath

**Affiliations:** 1 Discipline of Occupational Therapy, School of Medicine, Trinity College Dublin, Dublin, Ireland; 2 Discipline of Occupational Therapy, University of Sydney, Sydney, NSW, Australia; 3 Department of Occupational Science & Occupational Therapy, University College Cork, Cork, Ireland

**Keywords:** Emerging areas of practice, parenting, cross-sectional survey, Irish occupational therapy practice, assessment and intervention, barriers and solutions

## Abstract

**Introduction:**

Research on parenting with a disability is expanding. However, occupational therapy practices relating to parenting are poorly understood. This study explored the knowledge, confidence and practices of occupational therapists in Ireland supporting parenting roles.

**Method:**

A cross-sectional online survey, designed to address the study aims, was conducted using convenience and snowball sampling. Data were analysed with descriptive statistics and content analysis. Binary logistic regression examined factors influencing how often therapists working with adults who were parents (i) asked about and (ii) provided intervention related to parenting.

**Results:**

Ninety-seven therapists completed the survey. Most respondents reported that parenting was within their professional scope; however, engagement with parents regarding parenting concerns was limited. Experience significantly predicted frequency of asking about parenting (*p* = 0.046). Few therapists conducted formal parenting assessments or interventions routinely. Most use informal assessments, including parental self-report when assessing parenting concerns. Parenting interventions were sparse. Training significantly affected confidence in supporting parenting approaches and knowledge (*p* < 0.001) and providing emotional support and problem-solving (*p* = 0.002).

**Conclusion:**

Occupational therapists in Ireland acknowledge parenting as a valued occupation for their clients, however, routine support for parenting concerns is limited. Advancing practice in this area requires enhanced training and professional support to address parenting needs.

## Introduction

Parenting is a valued and demanding adult occupation that can present challenges for all parents, and these challenges may be compounded by illness, injury, or disability. Parenting is multifaceted, encompassing interconnected elements such as caregiving, nurture, education, play, financial security and protection (Codd et al., 2023; Hojatzadeh et al., 2015). It also involves a continuum of learning, shaped by experiences of self and others, on how to effectively nurture and guide children (Sethi, 2019). Parenting performance, engagement and satisfaction are influenced by the physical, emotional and social capacity, and overall wellbeing of a person. The fundamental right of parents to be the providers of support and nurture to their children is recognised nationally (Irish Statue Book, 2020) and internationally (UN, 2024; World Health Organization [WHO], 2024). Irish family law emphasises the importance of maintaining ongoing relationships between children and both parents (Irish Statue Book, 2020). Supporting parenting capacity helps parents to sustain relationships with their children, which enables them to contribute to their child’s well-being and development and have an active involvement in their life. However, ill-health and disability can result in restrictions in the performance of parenting tasks, and the unpredictability of ill-health trajectories can negatively influence experiences of parenting (Avrech Bar et al., 2021; Codd et al., 2023; Zelkowitz et al., 2013). Additionally, parents’ perceptions of their own physical and psychological health have been reported to impact parent-child interactions and relationships, which can diminish their overall parenting experience (Chew et al., 2019; Stransky et al., 2022; Zelkowitz et al., 2013).

Occupational therapy theory supports the view that participating in meaningful occupations is positively linked to health and well-being (Wilcock and Hocking, 2015) with parenting included as a domain of concern for practice (American Occupational Therapy Association [AOTA], 2020). Supporting participation in valued occupations across the lifespan is the fundamental goal of occupational therapy and the focus of occupational therapy interventions (AOTA, 2020). This goal is aligned with the WHO perspective on rehabilitation which has broadened to supporting those with illness or disability to participate in meaningful life roles such as parenting (WHO, 2024). Parents with disability have identified concerns and difficulties when engaging in and managing the interconnected elements of their parenting role from pregnancy, perinatally and throughout their parenting years (Chew et al., 2019; Honey et al., 2024). Parents have reported that they would like occupational therapy to address parenting difficulties using individualised and targeted approaches (Codd et al., 2023; Honey et al., 2024; Pituch et al., 2022, 2024). However, knowledge as to if, when, or how occupational therapists address parenting in practice is sparse (McGrath et al., 2024).

OT4Parenting is an international collaboration of researchers and clinicians interested in researching and advancing occupational therapy practice to support parents across diverse populations and clinical settings using a parent-centric focus (ot4parenting.org). This collaboration has taken one of the first steps to advancing the role of occupational therapy in parenting by undertaking a global survey of 42 countries to explore current occupational therapy practice, and attitudes of occupational therapists about addressing parenting with clients (Honey et al., 2025; McGrath et al., 2025). Findings from a sample of 1357 respondents indicate that internationally, many occupational therapists recognise the importance of parenting roles, with 43.1% (*n* = 586) frequently enquiring about parenting as an occupation of concern (McGrath et al., 2025). However, current international occupational therapy practice in addressing parenting concerns remains limited with 34.2% (*n* = 465) of respondents in the global study reporting that they frequently/very frequently address parenting concerns in routine practice (McGrath et al., 2025). Additionally, this global study identified that occupational therapists practicing in East Asia and the Pacific, North America, Europe and Central Asia and Latin America and the Caribbean were more likely to work with clients on parenting roles compared to their counterparts in the Middle East and North Africa. Variations were identified in the scope and selection of parenting assessments, with most respondents relying on informal methods or broad measures of occupational performance. Additionally, limited interventions specifically designed to support parents facing challenges in their parenting roles were identified in current international practice (McGrath et al., 2025). These authors highlight that occupational therapy related to parenting support is underdeveloped, influenced by gaps in training and confidence and service design agreements (McGrath et al., 2025). The development of occupational therapy parent-focused training and resources, assessment tools and intervention packages is recommended to strengthen occupational therapy practice in this area, alongside improvements in the scope and organisation of models for service provision (Honey et al., 2025; McGrath et al., 2025).

Honey et al. (2025) acknowledge that the international findings on occupational therapy practice related to parenting vary due to several factors, including the country of practice. In Ireland, contemporary parenting experiences are reported to be challenging due to the complexity of parenting demands, balancing work and parenting roles and a lack of resources and supports for parents (Geraghty, 2021; Hickey and Leckey, 2021). Irish parent support services are fragmented, spread across social care, healthcare and community organisations, and are often perceived as inadequate, with limited accessibility to resources, particularly for parents with disabilities (Hickey and Leckey, 2021). Irish researchers have highlighted an impairment-focused approach within healthcare, where the emphasis is primarily on disability in parenting rather than on supporting ability (Geraghty, 2021) and parents with disabilities in Ireland have reported a lack of tailored parenting supports, which is linked to increased emotional strain (Codd et al., 2023; Leitão, 2023).

A continuum of inclusive parent support services, ranging from preventative, universal supports to targeted interventions across pregnancy and childhood, has been recognised as important for all parents in Ireland, particularly in addressing the specific needs of parents with disabilities (Hickey and Leckey, 2021; Leitão, 2023). However, the scarcity of research on parenting support in Ireland highlights the need for more comprehensive studies to inform Irish policy and practice (Leitão, 2023). Timely access to evidence-based, practical, parent-focused support delivered by a trusted source has been identified as beneficial in supporting parents (Hennessy et al., 2020). Understanding how current parenting support services are provided in Ireland is an important first step in addressing gaps and improving the effectiveness of interventions to ensure they meet the needs of all families. The purpose of this paper is to explore Irish occupational therapy practice related to parenting with the aim of understanding:

- The knowledge and confidence of occupational therapists addressing parenting in Irish practice, and characteristics of parent clients on their caseloads
- How occupational therapists address parenting in their practice in Ireland
- The relationship between asking about parenting and addressing parenting concerns in occupational therapy practice
- Barriers and strategies to enhance how occupational therapists in Ireland address parenting roles

## Method

### Design

A cross-sectional multivariate study design was used. Data were gathered using an online anonymous survey. The survey was designed specifically for the OT4Parenting study, an international study exploring occupational therapy practice with parenting globally (McGrath et al., 2025). Survey content was developed through a process of review of literature on parenting as an occupation, cognitive interviews with experienced occupational therapists, and a detailed review of the survey draft by a panel of international occupational therapy academics to check for clarity, face validity and cultural appropriateness of questions in various contexts (McGrath et al., 2025). The survey used REDCap (Harris et al., 2009) and was hosted at the University of Sydney. It included both fixed choice and open-ended questions about participants’ demographic characteristics, clinical practice relating to parenting roles and occupations and experiences in addressing parenting. The survey contained between 45 and 103 items with branching and display logic used to tailor the survey according to participant responses. Mandatory questions required responses before participants could proceed to the next section, enforced using built-in REDCap validation features that prompted users to complete unanswered required items. For questions where non-response was acceptable, explicit options such as ‘prefer not to say’ or ‘unsure’ were provided to allow participants to skip items without interrupting survey flow. A copy of the survey is available in Supplemental Material. The instrument was designed to be completed in approximately 15 minutes. To protect participant privacy, no measures were taken to detect multiple submissions. However, this was not considered a significant risk, as the authors were unaware of any individuals or groups with a vested interest in influencing the results. All surveys from participants who confirmed they met the inclusion criteria were included in the analysis. Where participants discontinued the survey prior to completion, the remaining data were still included, resulting in a survey completion rate of 95.9%. The study design and reporting were guided by the Checklist for Reporting Results of Internet E-surveys (CHERRIES) (Eysenbach, 2012). A copy of the CHERRIES checklist (Eysenbach, 2012) can be found in Supplemental Appendix 1.

### Sample

Convenience sampling was used, and included occupational therapists who were currently working, or had worked in the previous 5 years, with adults between 16 and 64 years of age as primary clients across all clinical areas and populations. Ireland’s health regulator reports that 3509 occupational therapists are registered to practice (CORU, 2023) and the Irish national occupational therapy professional society reports a membership of 1700 (AOTI, 2024). Participants for this study were identified through their national occupational therapy professional society in Ireland. Members of the national occupational therapy professional body who had consented to receive research invitations were emailed a copy of the participant information leaflet and survey hyperlink. Additionally, snowball sampling was included, whereby participants could share the survey hyperlink with other relevant occupational therapists who may not be registered with their professional body. The study was also advertised through social media outlets used by occupational therapists, using the hashtag #everyparentmatters.

Ethical approval to conduct the research was obtained from the research ethics committee of the Education and Health Sciences Research Ethics Committee, University of Limerick, and the University of Sydney Human Research Ethics Committee. The online survey did not request any identifiable information from participants and included the participant information sheet as a mandatory first step. Consent was assumed upon completion and return of the anonymous questionnaire.

Data collection was completed between June and December 2023.

### Data analysis

Descriptive and inferential analysis were conducted using SPSS (version 29.01.0). For the purposes of binary ordinal analysis, regression and to address sparse data issues in some categories, the original five-category variable relating to the frequency with which participants asked about and addressed parenting roles and occupations was recoded into two categories. The categories were collapsed as follows: ‘Very frequently’ and ‘Frequently’ were recoded as ‘Frequently’; ‘Sometimes’, ‘Occasionally’ and ‘Rarely or Never’ were recoded as ‘Infrequently’.

Data collected from open-ended questions were analysed using interpretive content analysis (Drisko and Maschi, 2016), a structured approach that facilitates in-depth understanding of qualitative data (Bengtsson, 2016). In the first stage, all responses were read several times by the first author, who has experience in qualitative methods, to gain a comprehensive understanding of the data. Initial codes were generated inductively, capturing meaningful units of text related to the study aims. New data were continuously compared with existing codes and either added to them or used to create new codes as appropriate. Codes were iteratively refined through discussion with the full research team and grouped into broader categories and themes representing shared patterns across responses. In the second stage, all data were re-examined to ensure coding consistency and completeness, and to confirm that categories accurately reflected the dataset. This process resulted in cohesive themes reflecting occupational therapists’ knowledge, confidence and practices in addressing parenting, as well as their experiences supporting parent clients in Irish settings. Credibility and dependability were enhanced through researcher triangulation and maintenance of an audit trail documenting analytic decisions, while confirmability was supported through reflexive team discussions to minimise bias.

## Results

### Profile and knowledge of occupational therapists and characteristics of parent clients

#### Profile of sample

Table 1 presents the demographic and professional characteristics of the Irish sample (*n* = 97) as well as the parent clients on their caseloads. Most respondents were female (*n* = 96, 98.9%) and had been working as occupational therapists for more than 10 years (*n* = 64, 65.9%). There was a spread of therapists from inpatient, outpatient and community settings. A significant majority (92%, *n* = 89) indicated that they believed that addressing parenting was within their professional scope and many (*n* = 69, 71.1%) routinely asked about parenting status in their practice. Few respondents (*n* = 9, 9.2%) reported specific training in parenting assessment or intervention beyond their qualifying occupational therapy programme.

**Table 1.** Characteristics.

Sample characteristics	*n*	%
Occupational therapists		
Female	96	98.9
Male	0	0.0
Prefer not to respond	1	1.1
Years working as an occupational therapist		
Early career 0–5 years	14	13.41
Mid career 6–20 years	44	45.2
Senior >20 years	39	40.2
Received specific training in parenting assessment or intervention beyond qualifying occupational therapy programme		
Yes	9	9.2
No/can’t recall	88	90.6
Service type		
Public (government) health service	78	80.4
Non-government/charitable service	5	5.1
Private (for-profit) health service	3	3.1
Independent private practice	14	14.4
Other	3	3.1
Service context		
Inpatient	33	34.0
Outpatient	29	29.8
Community	42	43.2
Caseload characteristics		
Client presenting condition		
Physical disability	50	51.5
Neurological disorders	49	50.5
Chronic disease	44	45.3
Mental health challenges	31	31.9
Sensory disability	13	13.4
Intellectual disability	8	8.2
Developmental disability	7	7.2
No specific health condition or disability (e.g. social disadvantage)	4	4.1
Other	3	31
Percentage of adult clients who are parents of children (0–18 years old)		
0–20%	42	43.3
21–40%	15	15.5
41–60%	19	19.6
61–80%	12	12.4
81–100%	6	6.2
I don’t know	3	3.1
Methods used to identify clients’ parental status		
I collect this data for every client as part of routine assessment	69	71.1
I ask about parenting if a client has a condition/circumstance that might impact parenting	23	23.7
I find out about parenting if the client or another colleague brings it up	17	17.5
All clients in my service are parents	5	5.1
I would rarely be aware of a client’s parental status	0	0

Methods to identify clients’ parental status were explored and are presented in Table 1. All respondents reported that they were always aware of clients’ parental status, with the majority (*n* = 69, 61%) reporting that this metric was routinely collected during assessment.

There was a similar representation of mothers (*n* = 70, 72.2%) and fathers (*n* = 66, 68%) seen in occupational therapy services, although most respondents reported rarely or never working with young parents (<18 years old; *n* = 52, 53.6%), non-custodial parents (*n* = 52, 53.6%), parents without citizenship (*n* = 46, 47.4%) and those who identified as lesbian, gay, bisexual, transgender, intersex, queer, asexual or LGBTIQA+ (*n* = 45, 46.4%).

#### Knowledge and confidence relating to occupational therapy assessment and intervention for parenting

The majority of respondents (86%, *n* = 83) reported that they had no specific training relating to parenting as an occupation, however, a small number of respondents (9%, *n* = 9) identified post-qualification, skills-based workshops, short courses, one-off lectures or clinical supervision and multidisciplinary team continuous professional learning which they stated supported their knowledge and training in this area. Respondents identified variety in the focus of this learning and cited a mix of programmes including those focused on parent occupations (e.g. Occupational Performance Coaching) versus child developmental outcomes (e.g. Alert programme, play therapy) and those focused on specific clinical populations versus generic parenting programmes (Parent Plus, Incredible Years).

Outside of formal training in parenting occupations, respondents reported that their knowledge and understanding of parenting were informed by a variety of factors and included experiences of working with parents (89%, *n* = 86), personal experience of being parented (89%, *n* = 86) and personal experiences of being a parent (74%, *n* = 72). Most respondents (89%, *n* = 86) reported that their occupational therapy qualifying degree did not provide them with sufficient skills to confidently address parenting with clients, although 66% (*n* = 64) did attribute their qualifying degree as contributing to their knowledge of parenting occupations. Many respondents indicated that they utilised self-directed reading and research (63%, *n* = 61) and interactions with specialist services and other health professionals (63%, *n* = 61) to contribute to their knowledge base of this occupation.

#### Addressing parenting in Irish occupational therapy practice

Ascertaining parenting status in routine Irish occupational therapy practice was reported by many respondents who indicated that they ask adult clients who are parents about their parenting roles and occupations very frequently/frequently (*n* = 46, 47.4%), or sometimes/occasionally (*n* = 33, 34.0%).

However, variation was observed between asking about parenting roles, assessing parenting as an occupation in practice, and including parent-focused interventions in occupational therapy delivery with this cohort. Most indicated that they sometimes/occasionally (*n* = 44, 45.3%) or rarely/never (*n* = 40, 41.2%) worked with clients who are parents on their parenting roles or occupations compared with only a small proportion (*n* = 13, 13.4%) who reported that they did so very frequently/frequently.

A binary logistic regression was conducted to examine whether therapists’ years of experience predicted how frequently they asked parents about their needs. The model was statistically significant, χ^2^(1) = 3.98, *p* = 0.046, indicating that experience significantly contributed to predicting the frequency of asking about parenting. The model explained between 8.4% (Cox and Snell *R*^2^) and 11.4% (Nagelkerke *R*^2^) of the variance and correctly classified 63.9% of cases. Therapists with more years of experience were nearly twice as likely to frequently ask parents about their parenting needs compared to those with less experience (OR = 1.91, 95% CI [1.01, 3.61]).

Approaches to assessment of parenting were explored with consideration of frequency of employing formal and informal assessment methods (see Table 2). Most respondents (88%, *n* = 85) reported that they rarely/never use formal assessment tools when assessing parenting occupations. Of those who did use formal assessment tools (*n* = 12), the majority (*n* = 10) reported that they use general occupational assessments such as the Canadian Occupational Performance Measure. Informal assessment of parenting performance was reported by 69% (*n* = 67) and the majority of these (*n* = 64) cited self-report from parents as informing the assessment. Other informal assessment methods included observation of specific parenting skills and tasks (*n* = 23), reports from family members (*n* = 19) or health or social care professionals (*n* = 19) and observation of parent-child interactions (*n* = 16). Due to the highly skewed distribution of responses, particularly the low number of participants reporting using formal assessment tools, further statistical analysis, including subgroup comparison, was not completed.

**Table 2.** Approaches to assessment of parenting performance (*N* = 97).

Assessment method and frequency	*n*	%
Assessment of parenting roles using formal assessment tools		
Rarely/never	85	87.6
Sometimes/occasionally	7	7.2
Frequently/very frequently	5	5.1
Assessment of parenting roles using informal assessment tools		
Rarely/never	30	30.9
Frequently/very frequently	33	34.0
Sometimes/occasionally	36	37.1
Assessment tools used to evaluate parenting roles and occupations (*n* = 16)		
Standardised parenting assessment specific to my client population	1	6.2
Standardised parenting assessment for parents with disability or chronic condition	0	0
Standardised parenting assessment for general population of parents	0	0
Non-standardised assessment or checklist for parenting specific to my client population	3	18.7
Generic occupational therapy assessment, for example, COPM	10	62.5
Formal assessment methods (*n* = 28)		
Observation of parent-child interactions	2	7.1
Observation of specific parenting skills and tasks	5	17.8
Self-report by the parent	12	42.8
Reports from family members or other informal support networks	5	17.8
Reports from other health and social service professionals	4	14.3
Other	0	0
Informal assessment methods (*n* = 97)		
Observation of parent-child interactions	16	16.5
Observation of specific parenting skills and tasks	23	23.8
Self-report by the parent	64	65.9
Reports from family members or other informal support networks	19	19.6
Reports from other health and social service professionals	19	19.6

Respondents who indicated that they worked with parents more than ‘rarely/never’ were asked to think about the parents they had worked with on parenting issues and report the types of parenting interventions they provided. Their responses are presented in Figure 1 (*n* = 57). Within current occupational therapy delivery, respondents indicated that the most delivered parent-focused interventions include teaching parenting tasks and skills (*n* = 44, 77%), providing emotional support and/or problem-solving around parenting issues (*n* = 50, 88%) and referring to/supporting the parent to engage with community supports for parenting (*n* = 40, 70%). Individual support (*n* = 56, 98%) was the most reported mode of delivery for parent-centric interventions with a small number of responses indicating that they use structured individual parenting programmes (*n* = 3), and group programme for parents (*n* = 3).

**Figure 1.**
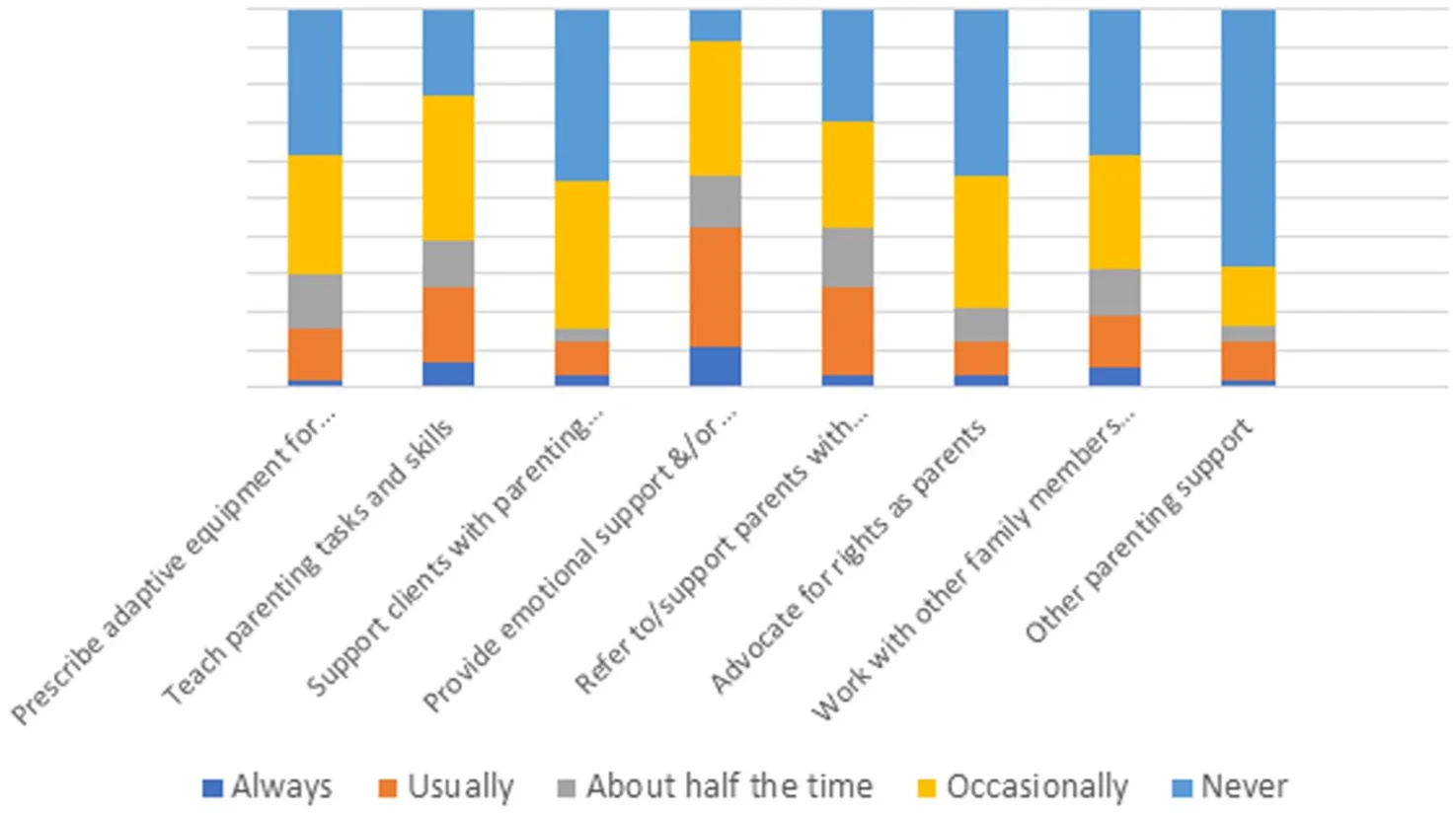
Frequency of use of types of interventions occupational therapists deliver to support parents with parenting tasks (*N* = 57).

To facilitate statistical analysis, frequency responses (*n* = 57) were collapsed into three categories: frequently (always/usually), infrequently (occasionally/about half the time) and never. These recoded variables were used to explore the association between prior training in parenting-related practice and reported frequency of intervention use. A series of chi-square tests were conducted to determine whether receiving specific training in parenting-related occupational therapy or years of experience were associated with more frequent use of parenting interventions. However, no associations remained statistically significant after applying the Bonferroni correction to adjust for multiple comparisons (adjusted alpha = 0.00625).

Open-ended questions invited respondents to share examples of occupation-focused parenting interventions. Qualitative findings highlighted the breadth of interventions across the parenting roles and how these were individualised and tailored to support parents with different clinical conditions.

‘Adapting activities like doing homework with your children if you have aphasia after a stroke or adapting position for breast feeding following a stroke’.

‘Working with parents with life limiting illnesses: I have worked with parents around leaving presents for children, e.g. arts and crafts items’.

‘Teaching self-management strategies and completing task analysis specific to parenting roles: Teaching how to “do differently”, e.g. managing bathing a small child in the context of pain, techniques to conserve energy in morning/school routine, navigating public transport or return to driving’.

‘I think part of the role I play with parents is supporting them to see what they do as “good enough” and to reduce the pressure they can put on themselves to parent brilliantly regardless of their own personal health needs’.

#### Confidence about assessing and addressing parenting

The majority of therapists reported being ‘not at all confident or comfortable’ in assessing parenting capacity/skills/performance (*n* = 69, 71.1%) or parenting confidence/satisfaction (*n* = 61, 62.8%). A smaller percentage expressed confidence in these areas, with only 4.1% (*n* = 4) feeling ‘very confident and comfortable’ in assessing parenting capacity/skills/performance and 8.2% (*n* = 8) in assessing parenting confidence/satisfaction.

A similar pattern of confidence emerged in relation to provision of occupational therapy interventions to address parenting concerns. A large proportion of therapists reported being ‘not at all confident or comfortable’ in interventions such as prescribing adaptive equipment for parenting activities (*n* = 34, 35.0%), teaching parenting tasks and skills (*n* = 55, 56.7%) and supporting clients with parenting approaches and knowledge (*n* = 78, 80.4%). Respondents reported more confidence and comfort in providing other, less direct interventions. For instance, 11.3% (*n* = 11) were ‘very confident and comfortable’ in providing emotional support and/or problem-solving around parenting issues, 9.3% (*n* = 9) in referring parents to community supports and 4.1% (*n* = 4) in advocating for parents’ rights.

Chi-square tests were conducted to examine the relationship between therapists’ training on parenting and their confidence and comfort in various parenting-related tasks. A post hoc Bonferroni correction was applied to adjust for multiple comparisons, resulting in an adjusted alpha of 0.0056. The results are presented in Table 3. After applying the Bonferroni correction for multiple comparisons, prior training in parenting was found to significantly affect therapists’ confidence in two areas of parenting related practice: supporting clients with parenting approaches and knowledge (*p* < 0.001) and providing emotional support and problem-solving around parenting issues (*p* = 0.002). However, training did not significantly affect confidence in other areas of parenting practice, such as assessing parenting skills, prescribing adaptive equipment, teaching parenting tasks, or advocating for parents’ rights.

**Table 3.** Occupational therapists’ confidence and comfort when delivering parenting assessments and interventions and influence of parent training (*N* = 97).

Parenting task/skill	Chi-square	df	*p*
Assessing parenting capacity/skills/performance	6.974	2	0.031
Assessing parenting confidence/satisfaction	2.954	2	0.228
Prescribing adaptive equipment for parenting activities	4.930	2	0.085
Teaching parenting tasks and skills (e.g. bathing, meal prep)	8.625	2	0.013
Supporting clients with parenting approaches and knowledge	17.495	2	<0.001*
Providing emotional support and/or problem-solving around parenting	12.541	2	0.002*
Referring to or supporting the parent to engage with community supports	10.220	2	0.006
Advocating for parents’ rights	6.069	2	0.048
Working with other family members (e.g. children, partners)	5.605	2	0.061

*For the purposes of analysis, response categories were collapsed to improve interpretability. Very frequently and frequently were combined and recoded as frequently, while sometimes occasionally and rarely or never were combined and recoded as Infrequently. A post hoc Bonferroni correction was applied to adjust for multiple comparisons, resulting in an adjusted alpha of 0.0056.

#### Barriers and strategies to occupational therapy practice supporting parenting

While most respondents agreed that parenting was within their scope of practice, overall confidence and engagement with parenting interventions did not reflect this. Qualitative data provided examples. One respondent shared:

‘I recently had a mild stroke (I’m in my early 40s). Neither the OT, nor any member of the MDT addressed this with me, despite me having significant caring responsibility as a parent of two young children and a carer for my resident elderly mother. I felt the initial OT assessment was a generic one that would be used for a typical 85-year-old stroke patient. . . . The issue of “returning to work” was discussed, but not how I would manage to parent . . . Nobody addressed my difficulties with parenting (due to fatigue and cognitive stress / overload). Luckily, as an OT myself and with OT friends I got this support, but I felt it should have been assessed / addressed by the OT /MDT’.

Most respondents (*n* = 87, 90%) identified the need to enhance the profession’s visibility and to expand occupational therapists’ role in supporting parenting. Respondents identified barriers in current occupational therapy practice routinely addressing parenting due to a lack of knowledge or training to adequately address parenting (*n* = 54, 55.7%); lack of parenting assessments (*n* = 43, 44.3%) and a lack of resources for occupational therapy interventions (*n* = 41, 42.3%).

Qualitative comments highlighted the influences of time constraints, acute short-term settings for occupational therapy intervention, lack of a clear pathway to assess and treat parent-centric difficulties, gaps in the occupational therapy role and the view that parenting support is desirable rather than core occupational therapy practice.

‘Parenting is not always a key priority – focus is on preparing for return home’.

‘Parenting is not main issue (for referral to OT) but one of many difficulties so prioritising helping parent manage their pain control, pace and prioritise assists them be better at coping with parenting’.

‘Lack of knowledge of the role of OT and the potential for OT to support parents. Parenting specifically is not something the MDT (multidisciplinary team) would refer to OT for. General coping/routine etc. would be more likely with parenting mixed in there as a consequence’.

Furthermore, respondents reported that the Irish healthcare system has a child-centric rather than parent-centric approach, which is a current barrier to supporting parents with disabilities and challenges.

‘I value the time required to talk and listen to parents’ experience. Unfortunately, this is not currently valued in the Irish public system. The pressure is on, so the intervention focuses on the child rather than parental well-being and parenting role’.

Open-ended questions invited respondents to suggest strategies to improve how occupational therapy services address parenting in routine practice. Qualitative findings highlighted opportunities to increase awareness and expectations that parenting falls within the scope of occupational therapy and to enhance role definition within occupational therapy and across broader practice settings. Upskilling occupational therapists to address parenting as an occupation was commonly reported, emphasising the need for increased training, resources and knowledge in parenting assessments and interventions. Respondents highlight that this could be achieved through CPD skill enhancement and postgraduate learning initiatives. Additionally, the need to invest in the Irish occupational therapy workforce across public health, social and medical healthcare models was identified. Finally, respondents emphasised the importance of an increased research focus on parenting as an occupation and on occupational therapy interventions to evaluate their impact.

## Discussion and implications

This study is the first to examine the knowledge, confidence and practices of occupational therapists in supporting parenting roles within an Irish context. While therapists in our study widely recognised parenting as a meaningful occupation, our findings suggest that parenting concerns are not routinely assessed or addressed in practice. This is consistent with international data, which also highlight gaps in the systematic integration of parenting into occupational therapy service delivery (Honey et al., 2025; McGrath et al., 2025; McGrath et al., 2024). Therapists in our study were more likely to ask about parenting when they had greater professional experience. However, this finding should be interpreted with caution, as years of experience may be confounded with age and personal life stage. In the Irish context, where occupational therapy education more typically occurs at undergraduate level, older therapists are likely to have both more professional experience and a higher likelihood of being parents themselves. These factors may influence their comfort, awareness and perceived relevance of addressing parenting in practice. While the cross-sectional design does not allow us to infer causality, it may be possible that experienced therapists adopt a more holistic view of client roles. Alternatively, therapists who are parents themselves may be more attuned to parenting issues. Although personal experience can enhance empathy, over-reliance on it may introduce bias or lead to inconsistencies in practice, particularly when not supported by professional training or evidence-based frameworks.

Our findings may also reflect some of the broader challenges experienced within Irish parenting support services, including fragmentation and a lack of universal, inclusive approaches (Geraghty, 2021; Hickey and Leckey, 2021). Most therapists surveyed were employed in healthcare settings, suggesting a potential gap in occupational therapy involvement within social care and community-based parenting support. There may be opportunities for the profession to expand its role in these areas, particularly in the context of early intervention and prevention.

Importantly, our findings suggest that while therapists in our study may ask about parenting roles, this is not consistently followed by formal assessment or intervention. This highlights a gap between recognising parenting as important and addressing it meaningfully in practice. International data indicate higher rates of formal parenting assessments and interventions (McGrath et al., 2025; McGrath et al., 2024), suggesting that Irish practice may benefit from further development in this area. Therapists in our study identified the lack of formal assessment tools as a barrier to effectively addressing parenting concerns. However, it remains unclear whether suitable tools are unavailable, underutilised, or simply unknown to practitioners. Clarifying this could inform future research and international collaborative efforts to develop or adapt assessment tools that better integrate parenting into routine occupational therapy practice.

Training emerged as a significant factor influencing confidence in delivering parenting-focused interventions, particularly those involving emotional support and problem-solving. However, confidence in other key areas, such as assessing parenting skills, prescribing equipment, or advocacy, remained low and was not significantly influenced by training. This may indicate a need for training programmes that are more comprehensive and practice oriented. Despite the limited use of structured parenting interventions, qualitative responses highlighted examples of valuable practices, including emotional support, skill-building and signposting to community resources. These align with existing research that suggests occupational therapists can meaningfully support a range of parenting roles, including nurturing, caregiving, play, advocacy and education (Codd et al., 2023; Sethi, 2019; Walker et al., 2016). However, the low frequency of such interventions in our sample suggests that these practices are not yet widely embedded in routine care, although this does not necessarily reflect the extent of unmet need. Of note, many therapists reported that their professional training did not prepare them adequately to address parenting. This is consistent with global findings (Honey et al., 2025; McGrath et al., 2025) and suggests a need for ongoing professional development and stronger inclusion of parenting within occupational therapy education. Developing a more structured, evidence-informed, and person-centred approach may help therapists feel more confident in addressing parenting roles.

While our findings highlight areas of opportunity, they should be interpreted with caution. Recruitment was limited to practitioners who had expressed willingness to receive research invitations, and convenience and snowball sampling were used. As a result, the sample may be biased towards those with a particular interest in parenting. The predominance of female respondents and the subset of qualitative responses also restricts generalisability. Overall, the sample represents only a small proportion of practicing occupational therapists in Ireland. Furthermore, the self-report nature of the survey may limit the accuracy of reported practice behaviours.

Future research should first examine existing assessment tools and parenting interventions to clarify what is currently available, underutilised or unknown to practitioners. This is an important next step before developing new resources and could inform international collaborative efforts to adapt assessment tools that better integrate parenting into routine occupational therapy practice. Subsequently, research should focus on the development and evaluation of tailored assessments and parenting interventions. Longitudinal studies could help determine the impact of training on therapist confidence and clinical outcomes. Partnerships between researchers and clinicians will also be essential to advance practice and support the inclusion of parenting within occupational therapy service design.

## Conclusion

Parenting is a typical adult role however, many parents with disability have reported challenges and concerns when engaging in and performing the interconnected elements of their parenting role (Honey et al., 2024; Lim et al., 2022). Our findings indicate that occupational therapists in Ireland recognise parenting as a valued occupation within their professional scope of practice, and the positive attitudes among Irish occupational therapists towards addressing parenting suggest a growing recognition of parenting as a meaningful occupation, aligning with McGrath et al. (2024) and the advocacy for a parent-centric approach. However, inclusion of parent-centric practice is limited in Irish practice. Embedding parent-focused approaches into routine occupational therapy services in Ireland requires multifaceted improvements, including research to develop and test tailored assessments and interventions for parents, as well as additional training and professional support for therapists.

**Key findings**

- Occupational therapists in Ireland consider parenting to be within their professional scope but often feel unprepared to address it in practice.
- Current occupational therapy support with parenting-related concerns is limited in routine service provision in Ireland.
- Advancing parent-centred practice requires the development of occupational therapy parenting assessments and interventions, along with training and support for therapists.

**What the study has added**

This study is the first to explore current parenting-focused practices in occupational therapy services in Ireland and highlights the barriers and potential solutions to provision of parent-centric occupational therapy services in Ireland.

## Supplemental Material

sj-docx-1-bjo-10.1177_03080226261418491 – Supplemental material for The role of occupational therapy in supporting parenting occupations: An Irish occupational therapy perspective Supplemental material, sj-docx-1-bjo-10.1177_03080226261418491 for The role of occupational therapy in supporting parenting occupations: An Irish occupational therapy perspective by Yvonne Codd, Anne Honey and Margaret McGrath in British Journal of Occupational Therapy

sj-pdf-2-bjo-10.1177_03080226261418491 – Supplemental material for The role of occupational therapy in supporting parenting occupations: An Irish occupational therapy perspective Supplemental material, sj-pdf-2-bjo-10.1177_03080226261418491 for The role of occupational therapy in supporting parenting occupations: An Irish occupational therapy perspective by Yvonne Codd, Anne Honey and Margaret McGrath in British Journal of Occupational Therapy
