# Supplementary material for: The role of occupational therapy in supporting parenting occupations: An Irish occupational therapy perspective
Source: Br J Occup Ther. 2026 Feb 15;89(10):676–85. doi: 10.1177/03080226261418491 (PMC13752425; doi:10.1177/03080226261418491)
Supplement: Supplementary file 1 [file sj-pdf-2-bjo-10.1177_03080226261418491.pdf]

Supporting parenting occupations: The role of occupational therapy

This is a survey about how OTs see the role of occupational therapy in supporting parenting occupations.

We need your thoughts regardless of whether or not you currently address parenting in your practice.

Please go to the information sheet for information about this study.

[Attachment: "Participant information statement - OT and parenting.pdf"]

I have read the information sheet and agree to take part in this survey.
- ◯ Yes
- ◯ No

As you have not consented to do the survey, please close your browser now.

Are you an occupational therapist?
- ◯ Yes
- ◯ No

Do you currently work as an OT with adults between 16 and 64 as primary clients?
- ◯ Yes
- ◯ No

In the last 5 years, have you worked as an OT with adults aged 16-64 as primary clients?
- ◯ Yes
- ◯ No

Great - you are eligible for this survey!
Even if you haven't addressed parenting with your clients, we want your views.

Thank you for your interest in this survey. As you are not an OT who has recently worked with adults as primary clients, you don't fit our eligibility criteria.

How would you describe your gender?
- ◯ Male
- ◯ Female
- ◯ Transgender
- ◯ Non-binary
- ◯ I use a different term
- ◯ I do not wish to answer

What is your preferred term?

____

For approximately how many years have you worked as an occupational therapist (including full time and part time)?
- ◯ 0-2 years
- ◯ 2-5 years
- ◯ 5-10 years
- ◯ 10-20 years
- ◯ More than 20 years

Have you had any specific training in parenting assessment/intervention beyond your OT qualifying degree (including short courses/workshops)?
- ◯ Yes
- ◯ No
- ◯ Unsure/can't recall

Please briefly describe the training. For example, what was the content, how many hours was the training, and whether there was some qualification attached.

____

About your role.

Please answer the following questions in reference to your most recent position working with adults 16-64 years old.

Please note: Questions relate to working with adults as primary clients, not working with families in relation to a child's condition.

Which of the following best describes the context of this role?
(please mark all that apply)

- ☐ Inpatient
- ☐ Outpatient
- ☐ Community

In what kind of organisation do/did you work in this role?
(please mark all that apply)

- ☐ Public (government) health system
- ☐ Non-government/charity organisation
- ☐ Private health system
- ☐ Private practice
- ☐ Other (please specify)

In what type of organisation do/did you work in this role?

____

What conditions do/did the people you work with in this role mainly experience?
(please select all that apply).

- ☐ Chronic disease
- ☐ Physical disability
- ☐ Sensory disability
- ☐ Intellectual disability
- ☐ Developmental disability
- ☐ Mental health challenges
- ☐ Neurological disorders
- ☐ No specific health condition or disability (e.g., social disadvantage)
- ☐ Other

What other conditions do people you work with mainly experience?

____

Please describe your clients (who they are and why you see them)?

____

In what country do/did you mostly practice in this role?

- ◯ Australia
- ◯ Canada
- ◯ Chile
- ◯ Hong Kong
- ◯ Israel
- ◯ Japan
- ◯ Jordon
- ◯ Lebanon
- ◯ Malaysia
- ◯ New Zealand
- ◯ Republic of Ireland
- ◯ Singapore
- ◯ South Korea
- ◯ Taiwan
- ◯ United Kingdom
- ◯ United States of America
- ◯ Other (Please specify)

In what country do/did you practice in this role?

____

What state, province or territory do/did you practice in this role?

____

How would you describe the area in which you work/ed in this role?<br>(you may mark more than one)	☐ Urban<br>☐ Rural<br>☐ Remote
More specifically, is/was this area:	☐ A capital city<br>☐ A regional centre
How would you classify your main clients' income level?	◯ High<br>◯ Medium<br>◯ Low<br>◯ Mixed<br>Malaysia and Israel only
What is the religious affiliation/lifestyle of the people you work with?	◯ Secular<br>◯ Traditional<br>◯ Religious<br>◯ Ultra-orthodox<br>Israel only
What is your highest occupational therapy qualification?	◯ Doctorate (OTD/professionals)<br>◯ Masters degree<br>◯ Bachelors degree<br>◯ Diploma

Your involvement in parenting.

Please answer the following questions in reference to your most recent position working with adults 16-64 years old as primary clients, that you described above.

Please note: "Parents" in this survey includes biological parents (custodial and non-custodial) and others acting in a parental capacity (e.g., guardians).

What percentage of your adult clients, approximately, were parents of children (0-18 years old)?	◯ 0-20%<br>◯ 21-40%<br>◯ 41-60%<br>◯ 61-80%<br>◯ 81-100%<br>◯ I don't collect this information/don't know
How did you most often find out whether or not a client was a parent?<br><br>(please mark all that apply)	☐ All clients in my service are parents<br>☐ I collect this data for every client as part of routine assessment<br>☐ I ask about parenting if they have a condition/circumstance that might affect parenting<br>☐ I find out if the client or another colleague brings it up<br>☐ I would rarely be aware of a client's parental status<br>☐ Other
What other way/s did you identify whether or not a client was a parent?	__________________________________
How frequently did you ask clients who were parents about their parenting roles and occupations?	◯ Very frequently<br>◯ Frequently<br>◯ Sometimes<br>◯ Occasionally<br>◯ Rarely/never

How frequently did you assess parenting roles with your clients using formal assessment tools?

- ◯ Very frequently
- ◯ Frequently
- ◯ Sometimes
- ◯ Occasionally
- ◯ Rarely/never

What sort of tools did you use to assess parenting with clients?
(please mark all that apply)

- ☐ Standardised parenting assessment specific to my client population
- ☐ Standardised parenting assessment for parents with disability or chronic conditions
- ☐ Standardised parenting assessment designed for general population parents
- ☐ Non-standardised assessment or checklist for parenting specific to my client population
- ☐ Non-standardised assessment or checklist designed for general population or other parents
- ☐ Generic occupational assessment (e.g., COPM)
- ☐ Other

What other types of tools did you use to assess parenting?

____

What method/s did the assessment tools you used involve?
(please mark all that apply)

- ☐ Observation of parent-child interactions
- ☐ Observation of specific skills and tasks
- ☐ Self-report from parents
- ☐ Reports from family members or other informal supports
- ☐ Reports from health and social services professionals
- ☐ Other

What other method/s did your formal assessments use?

____

How frequently did you assess parenting roles with your clients without using formal assessment tools?
(e.g., performance, goals, confidence, satisfaction)

- ◯ Very frequently
- ◯ Frequently
- ◯ Sometimes
- ◯ Occasionally
- ◯ Rarely/never

What did your informal assessment involve?
(please mark all that apply)

- ☐ Observation of parent-child interactions
- ☐ Observation of specific skills and tasks
- ☐ Self-report from parents
- ☐ Reports from family members or other informal supports
- ☐ Reports from health and social services professionals
- ☐ Other

What other method/s did you use for informal assessment?

____

How frequently did you work with parents on their parenting roles or occupations?

- ◯ Very frequently
- ◯ Frequently
- ◯ Sometimes
- ◯ Occasionally
- ◯ Rarely/never

What modes of intervention did you use to support parents with parenting?
(please mark all that apply)

☐ Structured individual parenting program
☐ Group programs for parents
☐ Individual interventions/support as required
☐ Other (please specify)

What other mode of parenting intervention do you provide?

____

*** You've told us that you have only occasionally, rarely or never assessed or addressed parenting with your clients. We are particularly interested in your thoughts and opinions around this area - thanks so much for continuing to complete this survey.

Thinking ONLY about clients you have worked with on parenting issues, how often have you done the following?

	Always	Usually	About half the time	Occasionally	Never
Prescribe adaptive equipment for parenting activities	○	○	○	○	○
Teach parenting tasks and skills (e.g., bathing, meal preparation, cyber-safety, managing routines)	○	○	○	○	○
Support clients with parenting approaches and knowledge (e.g., child/adolescent development, discipline strategies)	○	○	○	○	○
Provide emotional support and/or problem-solving around parenting issues	○	○	○	○	○
Refer to or support the parent to engage with appropriate community supports for parenting	○	○	○	○	○
Advocate for their rights as parents	○	○	○	○	○
Work with other family members (e.g., children, partners) to support parenting	○	○	○	○	○
Other parenting support	○	○	○	○	○

Please specify your other involvement with parenting.

____

How confident and comfortable do you feel about:

	Very confident and comfortable	Quite confident and comfortable	Not very confident or comfortable	Not at all confident or comfortable

Assessing parenting capacity/ skills/ performance	◯	◯	◯	◯
Assessing parenting confidence/satisfaction	◯	◯	◯	◯
Prescribing adaptive equipment for parenting activities	◯	◯	◯	◯
Teaching parenting tasks and skills (e.g., bathing, meal preparation, cyber-safety, managing routines)	◯	◯	◯	◯
Supporting clients with parenting approaches (e.g., child/adolescent development, discipline strategies)	◯	◯	◯	◯
Providing emotional support and/or problem-solving around parenting issues	◯	◯	◯	◯
Referring to or supporting the parent to engage with appropriate community supports for parenting	◯	◯	◯	◯
Advocating for the rights of parents	◯	◯	◯	◯
Working with other family members (e.g., children, partners) to support parenting	◯	◯	◯	◯

Thinking ONLY about clients you have worked with on parenting issues, how often have you supported parents to do the following?

	Always	Usually	About half the time	Occasionally	Never
Attend to the child's basic physical needs (e.g., feeding, bathing)	◯	◯	◯	◯	◯
Move the child (e.g., carrying, transporting)	◯	◯	◯	◯	◯
Provide materially for the child (e.g., appropriate food, clothing and school supplies, budgeting)	◯	◯	◯	◯	◯

Look after the child's health needs and keeping them safe (e.g., healthy choices, getting medical help, preventing and responding to safety issues)	○	○	○	○	○
Communicate with the child (e.g., verbal communication, observing and responding to child's needs)	○	○	○	○	○
Play and do leisure activities with the child	○	○	○	○	○
Provide the child with emotional support and stability (e.g., comforting, showing affection, consistency and predictability)	○	○	○	○	○
Teach the child or facilitate their learning (e.g., discipline, helping with schooling, teaching life skills, teaching emotion management)	○	○	○	○	○
Provide a supportive environment for the child (e.g., appropriate physical and social home environment and child-care arrangements)	○	○	○	○	○
Manage the child's social networks (e.g., providing opportunities to socialise, encouraging positive relationships)	○	○	○	○	○
Other (please specify)	○	○	○	○	○

Please describe the other parenting areas you address in your practice. ______________________________

Please describe the parenting interventions or supports you use most frequently in as much detail as you would like. ______________________________

Thinking ONLY about clients you have assessed or worked with around parenting issues, how old were the children?

	Always	Usually	About half the time	Occasionally	Never
Babies (0-1 year)	○	○	○	○	○
Toddlers (2-3 years)	○	○	○	○	○

Pre-schoolers (4-5 years)	◯	◯	◯	◯	◯
Middle childhood (6-11 years)	◯	◯	◯	◯	◯
Adolescents (12-18 years)	◯	◯	◯	◯	◯

Thinking ONLY about clients you have assessed or worked with around parenting issues, what types of parents were they?

	Always	Usually	About half the time	Occasionally	Never
Mothers	◯	◯	◯	◯	◯
Fathers	◯	◯	◯	◯	◯
Other primary caregivers (e.g., custodial grandparents, foster parents)	◯	◯	◯	◯	◯
Lesbian, gay, bisexual, transgender, intersex, queer, asexual and other sexually or gender diverse (LGBTIQA+) parents	◯	◯	◯	◯	◯
Non-custodial parents	◯	◯	◯	◯	◯
Young parents (< 18 years old)	◯	◯	◯	◯	◯
Parents from culturally and linguistically diverse backgrounds	◯	◯	◯	◯	◯
Foreign domestic workers (i.e., relating to parenting their own children overseas)	◯	◯	◯	◯	◯
Parents without citizenship	◯	◯	◯	◯	◯
Expats	◯	◯	◯	◯	◯
First nations parents	◯	◯	◯	◯	◯
Other groups of parents with specific needs	◯	◯	◯	◯	◯

Please describe the other specific group of parents that you worked with. ______________________________

Which of the following has prevented or reduced your involvement in addressing or exploring parenting occupations with parents you worked with?
(Please mark all that are relevant)

- ☐ It is not part of my position description
- ☐ Parenting is not part of the referral
- ☐ I do not see it as part of my role
- ☐ Lack of institutional/management support for addressing parenting
- ☐ I do not have time to address parenting
- ☐ I do not have enough knowledge or training to address parenting adequately
- ☐ I believe that parenting is best addressed by someone else with more specialised training
- ☐ My clients do not have parenting issues
- ☐ Lack of parenting assessments
- ☐ Lack of resources for OT interventions (e.g, programs, adaptive equipment)
- ☐ Other barriers
- ☐ Not applicable

Please further describe anything that has prevented or reduced your involvement in parenting assessment and/or support.

____

To what extent have the following contributed to your knowledge of parenting occupations?

	A great deal	Somewhat	A bit	Not at all	Not applicable
Occupational therapy qualifying degree	◯	◯	◯	◯	◯
Other formal training	◯	◯	◯	◯	◯
Interactions with specialist services or other health professionals	◯	◯	◯	◯	◯
Experience of working with parents	◯	◯	◯	◯	◯
Reading and research	◯	◯	◯	◯	◯
Personal experience of being a parent	◯	◯	◯	◯	◯
Personal experience of being parented	◯	◯	◯	◯	◯
Informal observation of other parents	◯	◯	◯	◯	◯
Other sources of knowledge (please specify)	◯	◯	◯	◯	◯

Where else do you get your knowledge of parenting?

____

Do you believe that parenting is within the occupational therapy scope of practice?

- ◯ Yes
- ◯ No
- ◯ Not sure

Do you feel that your OT qualifying degree provided you with sufficient skills to confidently address parenting with clients?

- ◯ Yes
- ◯ No

To what extent do you think that other occupational therapists who work with adults as primary clients address parenting in their practice?	◯ Frequently<br>◯ Sometimes<br>◯ Occasionally<br>◯ Rarely<br>◯ Never<br>◯ Not sure
In general, what do you think about the involvement and profile of occupational therapy in supporting parenting?	◯ It should be increased a lot<br>◯ It should be increased a bit<br>◯ It is about right as it is<br>◯ It should be decreased<br>◯ Not sure
What do you think is needed for occupational therapists to better support parenting for their clients?	____
Is there anything else that you would like to tell us about your involvement with supporting parenting or the overall profile of occupational therapy in supporting parenting?	____
