# Supplementary material for: The role of occupational therapy in supporting parenting occupations: An Irish occupational therapy perspective
Source: Br J Occup Ther. 2026 Feb 15;89(10):676–85. doi: 10.1177/03080226261418491 (PMC13752425; doi:10.1177/03080226261418491)
Supplement: Supplementary file 2 [file sj-docx-1-bjo-10.1177_03080226261418491.docx]

# Appendices

# Appendix 1 – CHERRIES Checklist (Eysenbach, 2012).

| **Checklist Item** | **Explanation** | **Page number** |
| --- | --- | --- |
| Describe survey design | A cross-sectional multivariate study design was used. Data were gathered using an online anonymous survey. The survey was designed specifically for the OT4Parenting study, an international study exploring occupational therapy practice with parenting globally. This study used a dual-sampling approach, combining convenience and snowball sampling to recruit participants from the target population. | 4 |
| IRB approval | Ethics Approval was obtained from ANONYMISED for REVIEW. | 5 |
| Informed consent | Participation in the study was voluntary. The online survey did not request any identifiable information from participants and included the participant information sheet as a mandatory first step. Consent was assumed upon completion and return of the anonymous questionnaire. | 5 |
| Data protection | Data privacy and confidentiality were ensured through anonymous participation, password-protected storage at REDCap and with adherence to the University of Sydney data protection policies | 4 |
| Development and testing | The survey was designed specifically for the OT4Parenting study. Survey content was developed through a process of review of literature on parenting as an occupation, cognitive interviews with experienced occupational therapists, and a detailed review of the survey draft by a panel of international occupational therapy academics to check for clarity, face validity and cultural appropriateness of questions in various contexts | 4 |
| Open survey vs closed survey | This survey was an open survey, allowing any eligible participants to take part without pre-selection. | 4 |
| Contact mode | Participants were identified through their national occupational therapy professional society in Ireland. Members of the national occupational therapy professional body who had consented to receive research invitations were emailed a copy of the participant information leaflet and survey hyperlink. Additionally, snowball sampling was included whereby participants could share the survey hyperlink with other relevant occupational therapists that may not be registered with their professional body. | 5 |
| Advertising the survey | The study was also advertised through social media outlets used by occupational therapists, specifically LinkedIn, and X using the hashtag #everyparentmatters. | 5 |
| Web/email | This web-based survey was hosted on REDcap, REDCap with adherence to the University guidelines. All data were downloaded from REDCap and imported into SPSS (Version 29.01.0). | 4 & 5 |
| Context | The survey was hosted on REDCap, a widely used online survey platform designed for academic research. Participants accessing the survey were directed through specific channels, such as email invitations or social media links. REDCap is a neutral survey platform, it does not inherently bias the sample. | 4 |
| Survey administration | The survey was voluntary. | 4 & 5 |
| Incentives | No incentives were offered for completing this survey. | 5 |
| Timeframe | Data collection in Ireland was completed between June and December 2023. | 5 |
| Randomisation of items or questionnaire | No randomisation of items or questionnaires was applied; all participants received the same question sequence. | Not reported in the main text |
| Adaptive questioning | Adaptive questioning was used to streamline the survey experience and minimise participant burden. Certain questions were conditionally displayed based on previous responses. | Not reported in the main text |
| Number of items | The number of survey items was between 45-103 depending on branching logic and was completed on a single page. | 4 |
| Number of screens | The survey was distributed over a single screen. | Not reported in the main text |
| Completeness check | Mandatory questions required responses before participants could proceed to the next section. This was enforced using built-in REDCap validation features, which prompted users to complete unanswered required items before moving forward. For questions where non-response was acceptable, explicit response options such as “prefer not to say” or “unsure” were provided to ensure participants could opt not to answer that questions but continue with the survey. | 4 |
| Review check | Respondents could edit their answers while still on the same page as the question. However, once they navigated to a new page of the survey, they were unable to review or modify their responses. This was clearly explained on the first page of the survey, where it was noted that any submitted data could not be retracted due to the anonymous nature of the survey. | Not reported in the main text |
| Unique site visitor | The number of unique site visitors was not computed to respect the privacy of the potential participants. Furthermore, it would have been possible for multiple occupational therapists to access the same computer in a workplace to complete the survey. Therefore, the calculation of the number of unique site visitors may not be an accurate indicator of the true number of potential participants who viewed the site. | Not reported in the main text |
| View rate (Ratio of unique survey visitors/unique site visitors) | The number of unique site visitors was not computed to respect the privacy of the potential participants. Furthermore, it would have been possible for multiple occupational therapists to access the same computer in a workplace to complete the survey. Therefore, the calculation of the number of unique site visitors may not be an accurate indicator of the true number of potential participants who viewed the site.  as the number of unique site visitors was not computed for the reasons outlined above. | Not reported in the main text |
| Participation rate (Ratio of unique visitors who agreed to participate/unique first survey page visitors) | The participation rate was not computed as the number of unique site visitors was not computed for the reasons outlined above. | Not reported in the main text |
| Completion rate (Ratio of users who finished the survey/users who agreed to participate) | A total of 1414 eligible participants commenced the survey and 1357 completed the survey giving a survey completion rate of 95.9% . This is reported in the partner paper (Honey et al. 2025; McGrath et al. 2025) | 4 |
| Cookies used  I.P. Check  Log file analysis | Strategies were not employed to detect the potential for multiple entries to protect the privacy of the participants. Furthermore, it was not expected that there would be attempts made at submitting multiple entries as the authors are unaware of any individuals or groups with a vested interest in the results of this study. | Not reported in the main text |
| Registration | Potential participants were not required to login to complete the study as it was an open survey | Not reported in the main text |
| Handling of incomplete questionnaire | All surveys were included in the analysis where the participant had confirmed that they met the inclusion criteria. Where participants discontinued from the study prior to completion they remaining data was still included in the analysis. | 4 |
| Questionnaires submitted with an atypical time stamp | The responses were not screened for an atypical timeframe as there was no incentive for completing the questionnaire. Therefore, it was expected that if participants did not wish to complete it, that they would discontinue prior to submission. | Not reported in the main text |
| Statistical correction | No statistical corrections were applied to adjust for the potential of the study to obtain a non-representative sample. | Not reported in the main text |
